# Physiological and molecular correlates of the screwworm fly attraction to wound and animal odors

**DOI:** 10.1038/s41598-020-77541-w

**Published:** 2020-11-27

**Authors:** Paul V. Hickner, Omprakash Mittapalli, Anjana Subramoniam, Agustin Sagel, Wes Watson, Maxwell J. Scott, Alex P. Arp, Adalberto A. Pérez de León, Zainulabeuddin Syed

**Affiliations:** 1 Department of Entomology, University of Kentucky, Lexington, KY 40546 USA; 2 USDA, Agricultural Research Service, Knipling-Bushland U.S. Livestock Insects Research Laboratory and Veterinary Pest Genomics Center, Screwworm Research Site, Pacora, Panama; 3 Department of Entomology and Plant Pathology, North Carolina State University, Campus Box 7613, Raleigh, NC 27695-7613 USA; 4 USDA, Agricultural Research Service, Knipling-Bushland U.S. Livestock Insects Research Laboratory and Veterinary Pest Genomics Center, 2700 Fredericksburg Rd., Kerrville, TX 78028 USA

**Keywords:** Zoology, Entomology

## Abstract

The screwworm fly, *Cochliomyia hominivorax* (Coquerel), was successfully eradicated from the United States by the sterile insect technique (SIT). However, recent detection of these flies in the Florida Keys, and increased risk of introductions to the other areas warrant novel tools for management of the flies. Surveillance, a key component of screwworm control programs, utilizes traps baited with rotting liver or a blend of synthetic chemicals such as *swormlure-4*. In this work, we evaluated the olfactory physiology of the screwworm fly and compared it with the non-obligate ectoparasitic secondary screwworm flies, *C. macellaria,* that invade necrotic wound and feed on dead tissue. These two species occur in geographically overlapping regions. *C. macellaria*, along with other blowflies such as the exotic *C. megacephala*, greatly outnumber *C. hominivorax* in the existing monitoring traps. Olfactory responses to *swormlure-4* constituents between sex and mating status (mated vs unmated) in both species were recorded and compared. Overall, responses measured by the antennograms offered insights into the comparative olfactory physiology of the two fly species. We also present detailed analyses of the antennal transcriptome by RNA-Sequencing that reveal significant differences between male and female screwworm flies. The differential expression patterns were confirmed by quantitative PCR. Taken together, this integrated study provides insights into the physiological and molecular correlates of the screwworm’s attraction to wounds, and identifies molecular targets that will aid in the development of odorant-based fly management strategies.

## Introduction

The larvae of the screwworm fly, *Cochliomyia hominivorax* (Coquerel, 1858), are obligate ectoparasites of warm-blooded animals, with a historic range of subtropical and tropical regions of North and South America, and parts of the Caribbean. Great economic losses from screwworm to livestock production in the United States, estimated to be US$ 70–100 million annually in the 1960s, inspired scientists with the U.S. Department of Agriculture (USDA) to develop the sterile insect technique (SIT) for the eradication of screwworm^1,2^. The *C. hominivorax* SIT program, considered as one of the most effective pest eradication programs ever conducted, has eradicated screwworm from all of North and Central America, as well as Curaçao, Puerto Rico, and an introduction to Libya^3^. Presently, a barrier zone on the border of Panama and Colombia is maintained and co-managed by the USDA and the Panamanian Ministry of Agriculture (COPEG). The program consists of *C. hominivorax* mass-rearing facility, aerial dispersal center, and an extensive network of field personnel that respond to probable cases, conduct outreach and training with local ranchers, and operate livestock transportation check stations.

Effective and integrated pest management strategies depend on a strong understanding of the population dynamics. Blood feeding and parasitic insects display robust olfactory driven behaviors^4,5^, and exploiting the sense of smell (olfaction) is increasingly appreciated in developing the monitoring and management tools for forensic^6^ and medically important dipteran flies^7^. Routine testing of sterile *C. hominivorax* field performance and capture of wild flies in outbreak or in non-eradicated areas utilize two trapping methods: vertical sticky traps baited with *swormlure-4*^8^, or putrefied beef liver^9^. Development of these trapping systems evolved over the course of the screwworm eradication program spanning several decades. Early observation that *C. hominivorax* females were attracted to rotting liver^10^, animal wounds^11^, bacterial contaminated bovine blood^12^, and fluid from sheep wounds infested with larvae^13^ led the way for isolating and identifying chemical constituents from these natural substrates. Chemical analyses of one such attractive oviposition substrate, incubated blood, identified phenol, 4-methylphenol (*p*-cresol) and indole as major constituents^14^. Testing 35 compounds implicated as breakdown products of protein and fatty acids resulted in the development of a synthetic attractant blend, later called *swormlure*, that proved 5.7 times more effective to males and reduced the trapping of non-target species capture by *ca*. 87% in comparison with beef liver^15^. This blend was further improved by adding dimethyl disulfide and removing acetone, becoming *swormlure-2*^16,17^. Due to environmental challenges as the screwworm eradication program pushed farther south into Mexico, *swormlure-2* was not as effective. An adjustment to the release rates of the constituent compounds resulted in *swormlure-4*, the currently used attractant^8^.

A series of elegant experiments by Cork et al., starting with the chemical and electrophysiological analysis of the natural larval infested wound fluid (NWF) identified an array of electrophysiologically active compounds^18^. These compounds were subsequently evaluated and compared with the sentinel sheep, NWF and *swomlure-4*^19^, and offered mixed results, especially in terms of sex ratios in trap captures. This study therefore highlighted the need for future research to account for the physiological responses from males and female of all reproductive status^19^. Therefore, despite years of research into the chemical ecology of adult *C. hominivorax* towards developing a reliable and robust synthetic bait, routine surveillance of myiasis cases in the barrier zone and endemic areas still rely largely on liver bait stations and *swormlure-4*^3^ as was recently demonstrated following the outbreak in Florida^20^. This points to the need for more comprehensive research strategies combining physiological and molecular analyses to identify robust and reliable baits.

In insects, physiological sensitivity and behavioral response to odors vary depending on the organism’s physiological state such as feeding and nutritional status, mating status and sexual maturation^21^, as demonstrated in flies^22,23^, moths^24^ and mosquitoes^25^. Screwworm flies are no exception. Male *C. hominivorax* do not need to feed on an animal protein source, thus male attraction to the compounds in *swormlure* is likely in search of females searching for an oviposition site or mimicking flowers that emit carrion odors^26^. In the females, responsiveness to odors such as those that indicate the nutritive or reproductive resources is modulated by oocyte maturation^27^. Additionally, different odor sources attracted females at different stages of ovarian development^28^. Whereas *swormlure-4* is assumed to be equally attractive to nulliparous and parous *C. hominivorax* females, liver traps were most attractive to parous females likely looking for protein meals rather than oviposition sites^15,16^. In terms of sex differences in capture rates, *swormlure-4* has been reported to attract an equal number of males and females^19,29^, or biased towards females^8^.

We addressed these intriguing questions by employing a multipronged strategy. First, we measured the olfactory sensitivity of *C. hominivorax* males, virgin or gravid females to the constituent compounds of *swormlure-4*. Next, leveraging the newly sequenced genome assembly of this fly^30^, we isolated and analyzed the antennal transcriptome by RNA-Sequencing to gain insights into the molecular basis of olfaction in male and female screwworm flies. Antennal olfactory sensitivity is the outcome from the binding of odorants with the members of three divergent protein families—odorant receptors (ORs), ionotropic receptors (IRs), and gustatory receptors (GRs)—together referred as chemosensory gene families^31^. We previously identified 79 ORs, 84 GRs, 88 IRs and 51 odorant binding proteins (OBPs) in the screwworm genome^30^. The birth and diversification of these gene families^32^, together with the functional characterization of candidate genes—such as those differentially expressed between sexes, or physiological stages of female—offers the potential to isolate and identify chemicals that are highly effective and selective^4^. Taken together, this work identifies the olfactory similarities and differences between two screwworm species and provides insight into the molecular phylogeny of chemosensation in the screwworm flies.

## Results

### Olfactory responses to *swormlure* constituents

**Figure 1:**
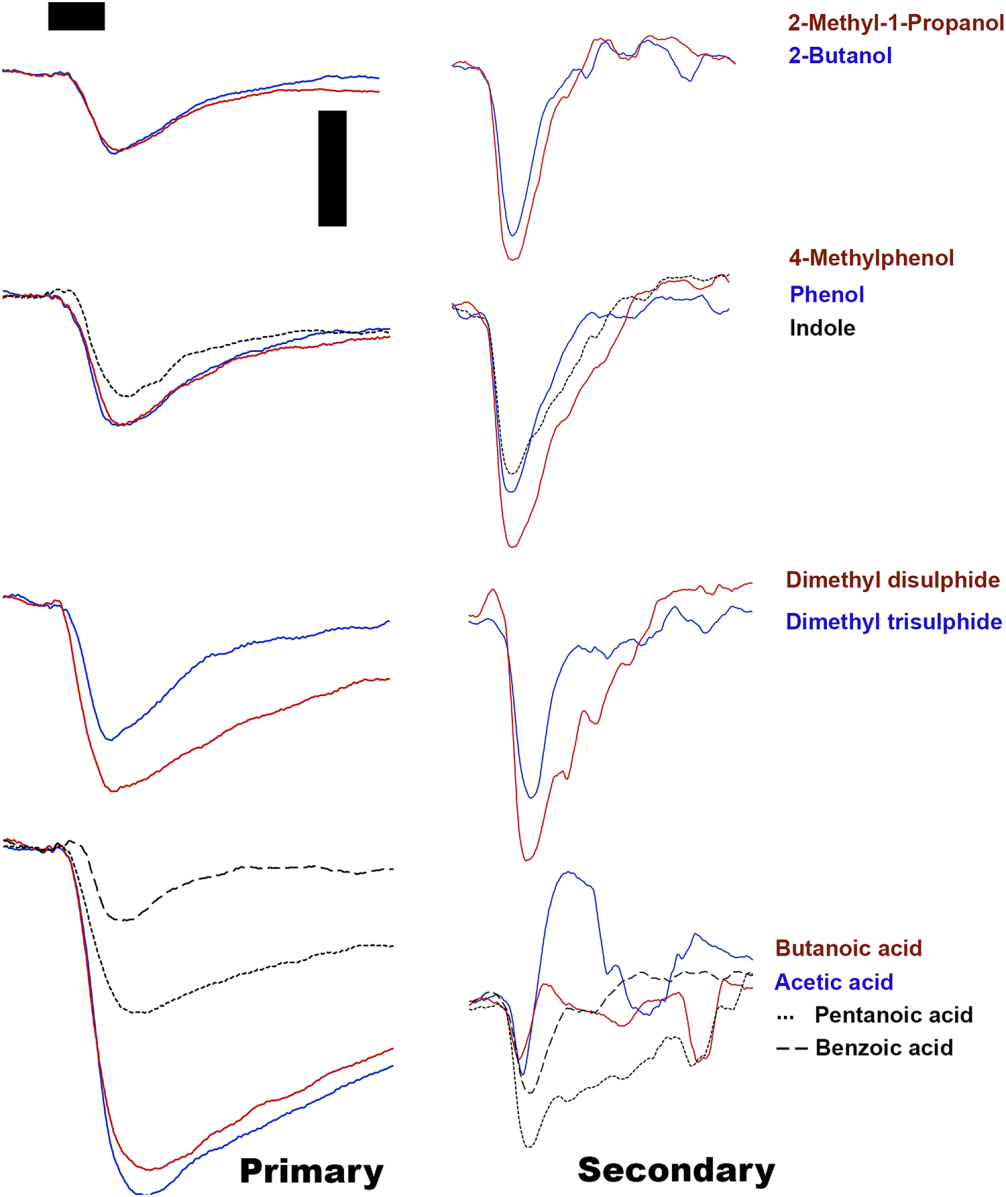
Antennal responses of the primary (left) and secondary screwworm flies to the constituent odorants of *swormlure-4* as measured by electroantennography. Traces are typical EAG signals depicting downward deflections, except acetic acid and butanoic acid traces from primary screwworm flies. Scale bars are 500 ms (horizontal; also represents the onset of the stimulus delivery) and 1 mV (vertical).

**Figure 2:**
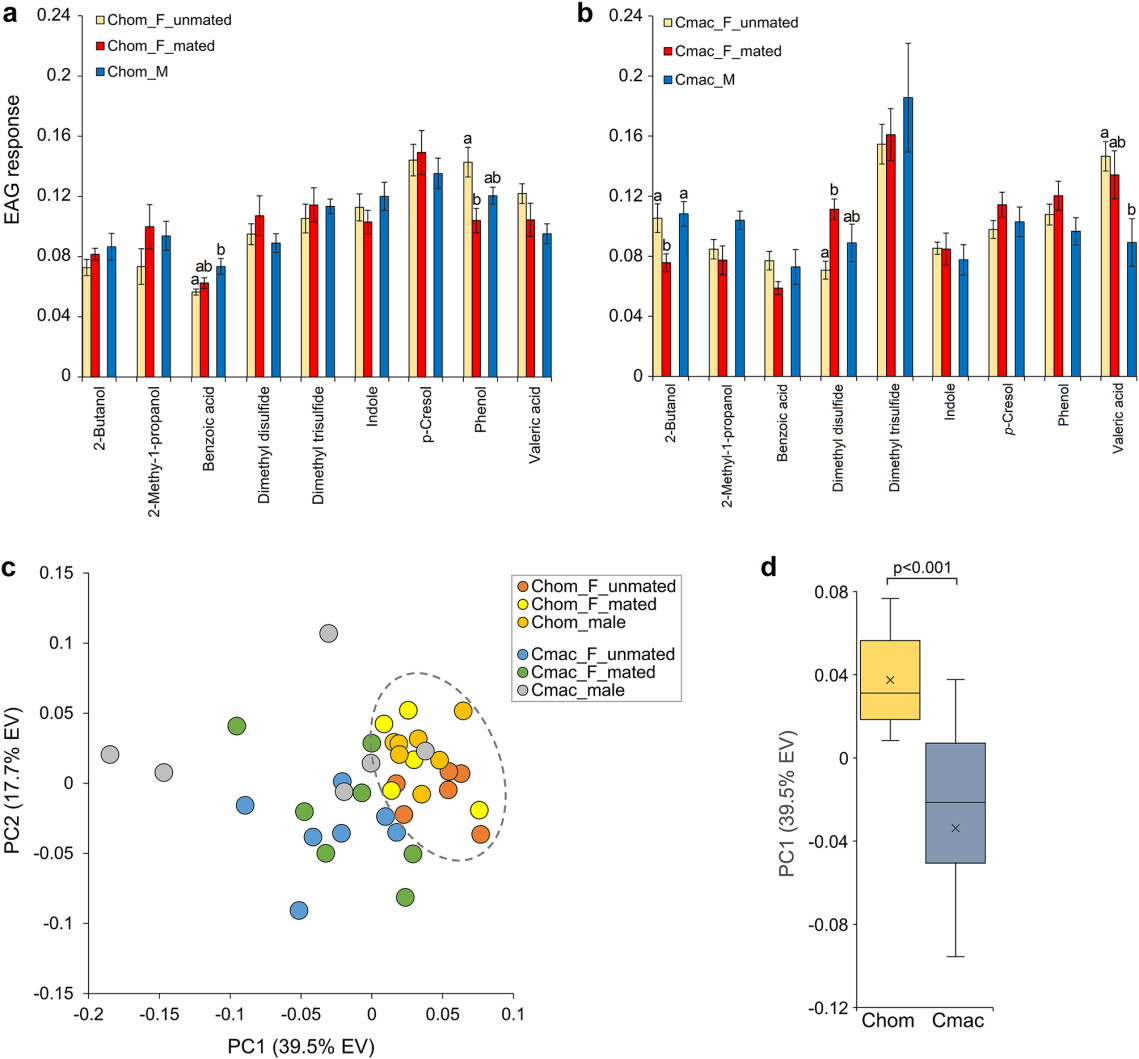
Antennal response by the primary (*C. hominivorax*) and secondary (*C. macellaria*) screwworm flies to components of *swormlure-4*. (**a**) Benzoic acid elicited a significantly larger response from male *C. hominivorax* compared to unmated females, while phenol elicited a larger response from unmated females compared to mated females. Significance (indicated by different letters above the columns in (**a**, **b**) based on one-way ANOVA and Tukey’s post-hock tests (*p* < 0.05). (**b**) 2-Butanol elicited a smaller response in mated female *C. macellaria*, while dimethyl disulfide elicited a larger response from mated compared to unmated females. Valeric acid elicited a larger response from unmated females compared to males. (**c**) Principal component analysis based on the antennal responses largely separated *C. hominivorax* and *C. macellaria*. (**d**) Species were significantly different based on Mann–Whitney rank sum test of PC1 component scores.

Antennal response of *C. hominivorax* and *C. macellaria* to the components of *swormlure-4*^8^ were measured using the electroantennogram (EAG) technique. In addition, we tested the response of dimethyl trisulfide, a recently described attractive odorant for the screwworm^33^. These compounds elicited robust and highly reproducible EAG responses in both species (Fig. 1). Two compounds, acetic acid and butyric acid, elicited both hyperpolarization and depolarization in both species and therefore were omitted from subsequent statistical analyses. Majority of the antennal responses to tested chemicals—six of the nine—were not significantly different between sexes, or females’ mating status. However, antennal response by *C. hominivorax* to two compounds, benzoic acid and phenol, differed between sex and/or mating status (Fig. 2a). Response of unmated females to benzoic acid was smaller than that of males (p = 0.023), while the response of unmated females to phenol was larger than that of mated females (p = 0.012) (Fig. 2a).

The antennal response of *C. macellaria* to three compounds—2-butanol, dimethyl disulfide and valeric acid—differed among sex and/or mating status, out of the 9 chemicals tested (Fig. 2b). The response of *C. macellaria* unmated females and males to 2-butanol was larger than that of mated females (p = 0.023), while the response of unmated females to dimethyl disulfide was smaller than that of mated females (p = 0.023), and the response to valeric acid was larger in unmated females compared to males (p = 0.028) (Fig. 2b). Principal component analysis (PCA) of antennal responses to the nine compounds listed in Fig. 2a and b separated *C. hominivorax* and *C. macellaria* by PC1 (p ≤ 0.001, Fig. 2d); however, intraspecies differences based on sex and/or mating status were not obvious in the PCA plot (Fig. 2c). PC1 captured the most variation (39.5%). The top three PC1 loading factors (weighing negatively) were 2-methyl-1-propanol, 2-butanol and dimethyl trisulfide, and the top three positively weighing compounds were indole, *p*-cresol and phenol.

### Male and female antennal transcriptomes of *C. hominivorax*

**Figure 3:**
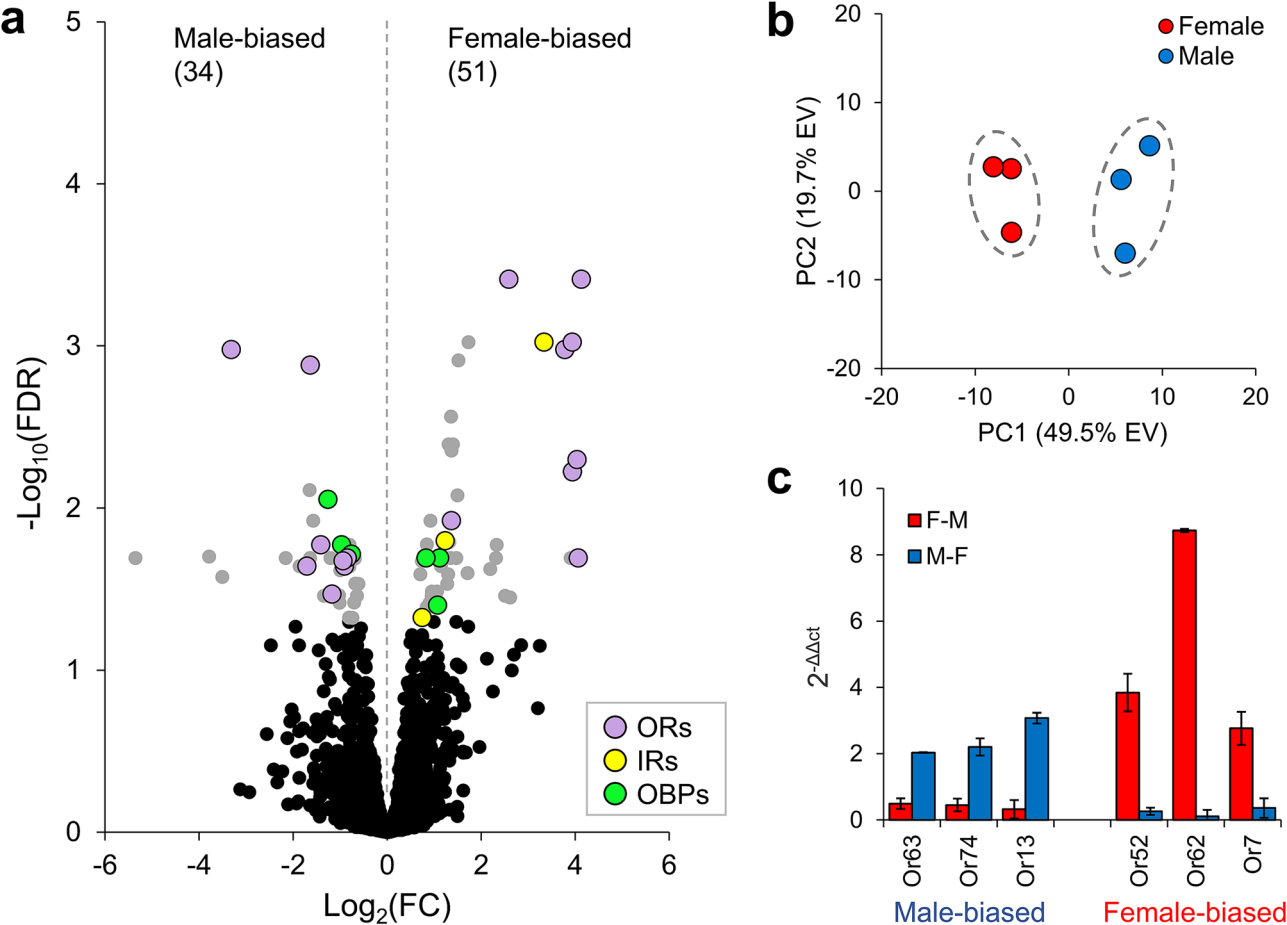
Quantitative analysis of the antennal transcriptome in male and female *C. hominivorax*. (**a**) A total of 10,442 genes were expressed in the antennae, of which 85 were differentially expressed (DE) between sexes (FDR corrected *p* < 0.05). Thirty-four genes showed male-biased expression and 51 showed female biased expression. Of the 85 DE genes, 27 were chemosensory genes (16 ORs, 0 GRs, 3 IRs and 8 OBPs). (**b**) Multivariate analysis was used to explore sex-specific differences in 114 chemosensory genes expressed in the antennae. PC1 explained 49.5% of the variation and grouped the samples according to sex. (**c**) RT-qPCR validation of six olfactory receptor genes differentially expressed between male and female *C. hominivorax* antennae. Data is the average of the biological replicates, and error bars illustrate standard error of the mean (SEM). Expression levels of each of the six genes were significantly different (p < 0.05).

A total of 10,442 genes were expressed in the antennae (Supplemental file 1), of which 114 were annotated as chemosensory genes (55 ORs, 15 GRs, 21 IRs and 23 OBPs). It is worth mentioning that these numbers are lower than those we reported in whole genome annotation^30^, and simply reflect the fact that some of these genes are expressed in other tissues such as legs and mouth parts, and/or at different developmental stages. A total of 85 genes were differentially expressed (DE) between the two sexes (Fig. 3a): 34 were higher in males (male-biased) and 51 were higher in females (female-biased). Twenty-seven of the 85 differentially expressed genes (31.8%) were chemosensory genes (16 ORs, 3 IRs and 8 OBPs) (Fig. 3a). We next explored sex-specific differences in chemosensation by conducting principle component analysis (PCA) of all 114 chemosensory genes. PCA based on gene expression of the chemosensory genes showed a clear separation of the sexes by PC1, which explained 49.5% of the variation (Fig. 3b). Validation by qRT-PCR using independently isolated RNA of six DE ORs—three each male-biased (*ChomOr63*, *ChomOr13* and *ChomOr74*) and female-biased (*ChomOr52*, *ChomOr62* and *ChomOr7*)—expression corroborated results based on RNA-seq (Fig. 3c).

**Figure 4:**
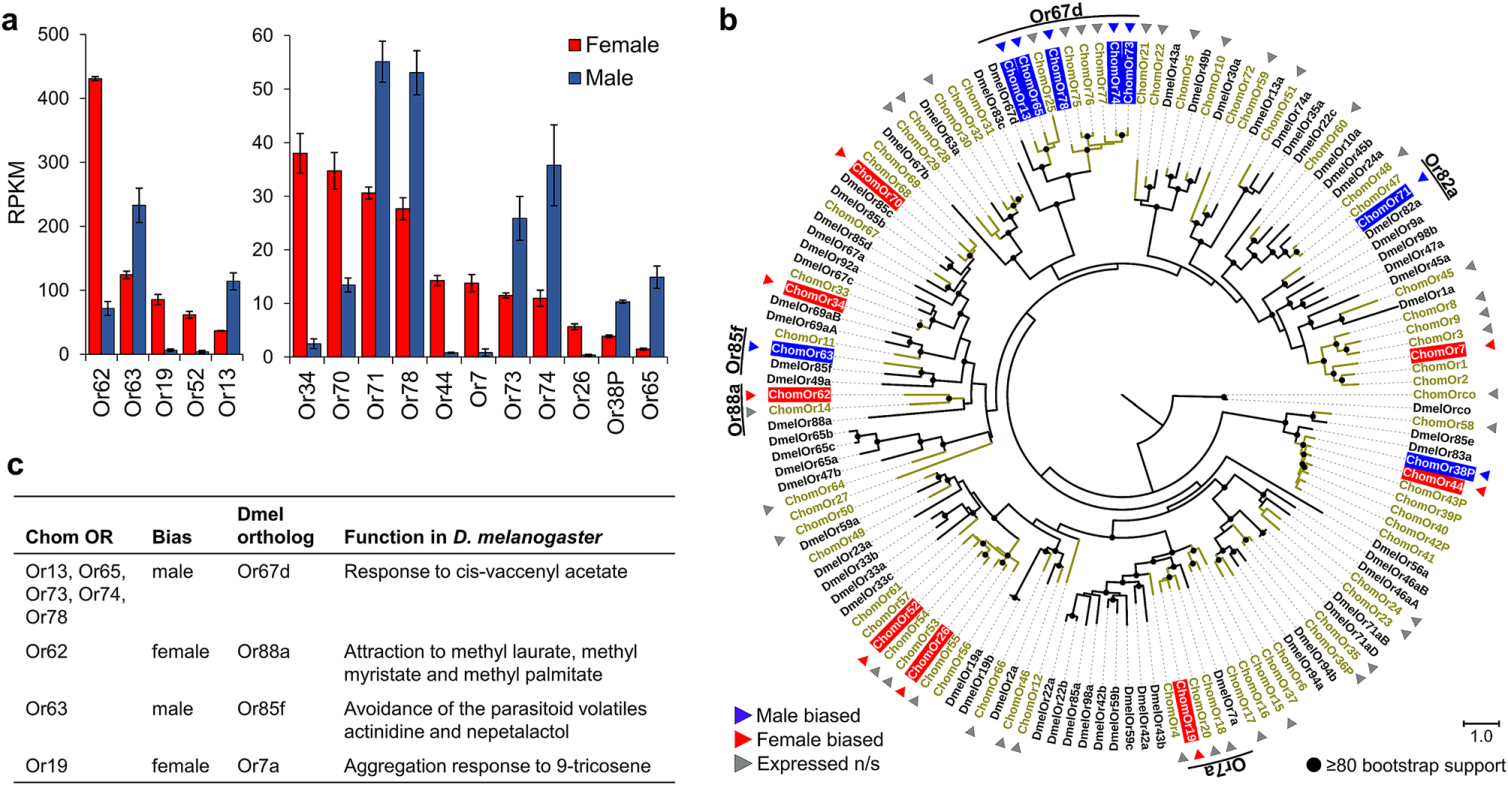
Sex-biased expression of ORs in *C. hominivorax* antennae. (**a**) Fifty-five ORs were expressed in the antennae, of which16 were differentially expressed between males and females, 8 each in females and males. Differential expression based on FDR adjusted p < 0.05. (**b**) Nine *C. hominivorax* ORs are closely related to *D. melanogaster* ORs based on the estimated OR phylogeny. Figures were generated using the interactive Tree of Life (iTOL) v4 software^36^ (https://itol.embl.de/). (**c**) Five male-biased ORs are closely related to the *cis*-vaccenyl acetate (cVA) pheromone receptor (Or67d) in *D. melanogaster*^34,35^, while one (*Or63*) is closely related to *Or85f*, which has been implicated in avoidance of the parasitoid volatiles actinidine and nepetalactol^37^. Two of the female-biased ORs are closely related to the *9*-tricosene (Or7a), and methyl laurate, methyl myristate and methyl palmitate (Or88a) receptors in *D. melanogaster*^38,39^, all of which are implicated as fly pheromones.

**Figure 5:**
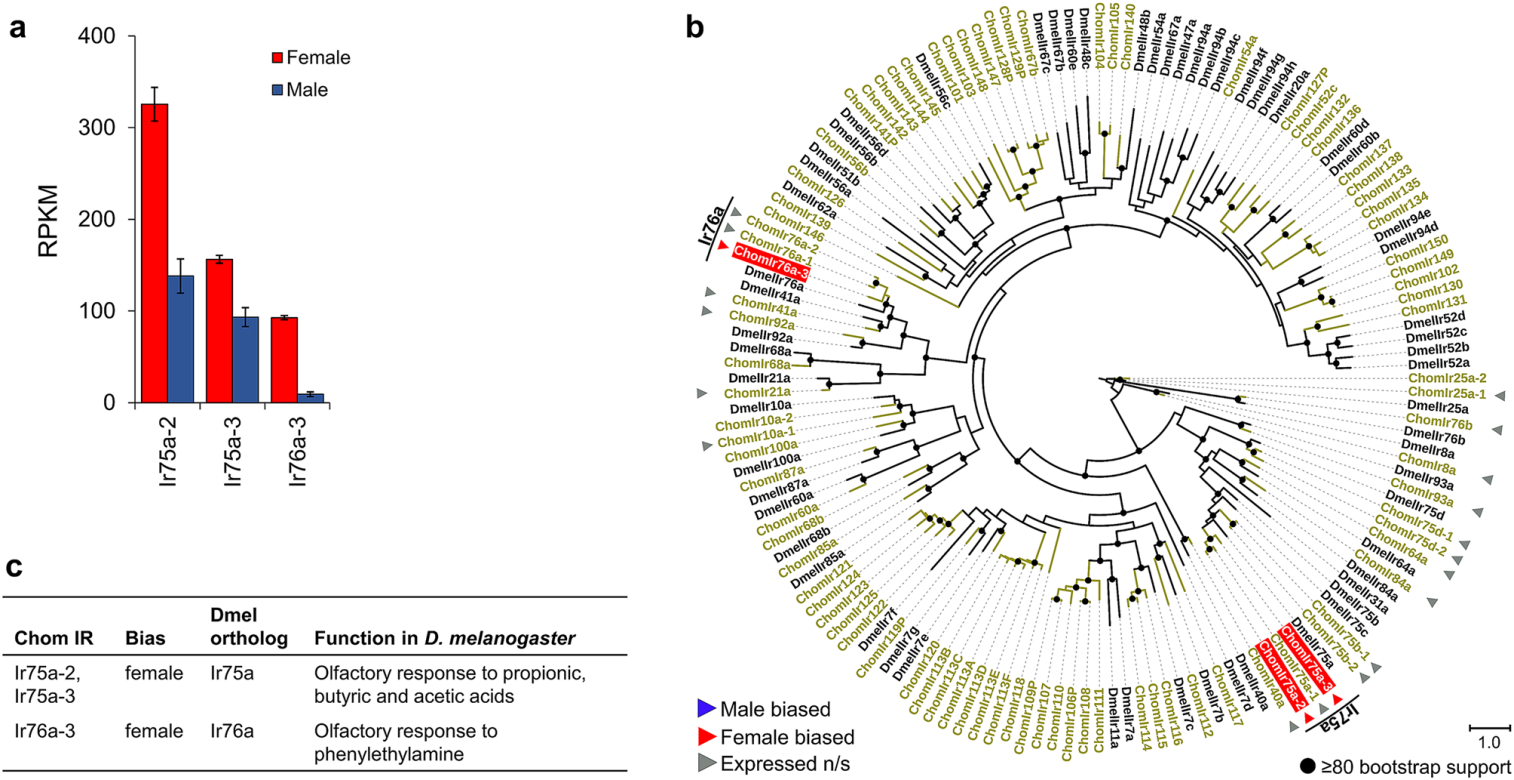
Sex-biased expression of IRs in *C. hominivorax* antennae. (**a**) Twenty-one IRs were expressed in the antennae, of which three were differentially expressed (DE) between sexes, all with higher expression in females. Differential expression based on FDR adjusted *p* < 0.05. (**b**) The three DE IRs are related to two *D. melanogaster* receptors based on the IR phylogeny. Figures were generated using the interactive Tree of Life (iTOL) v4 software^36^ (https://itol.embl.de/). (**c**) In *D. melanogaster*, Ir75a confers an olfactory response to propionic, butyric and acetic acids^40^, while Ir76a elicits an olfactory response to phenylethylamine^41^.

A total of 55 ORs were expressed in both sexes, with the exception of *Or3,* which had no read counts in any of the male replicates. Of the 55 ORs expressed in the antennae, 16 (29.1%) were DE, with eight displaying female-biased expression and eight displaying male-biased expression (Fig. 4a). Based on the estimated OR phylogeny, three female-biased and six male-biased ORs were closely related to *D. melanogaster* ORs (Fig. 5b). Five ORs with male-biased expression (*ChomOr13*, *ChomOr65*, *ChomOr73*, *ChomOr74* and *ChomOr78*) are in a clade with *DmelOr67d*, the *cis*-vaccenyl acetate pheromone receptor^34,35^ (Fig. 4b,c).

**Figure 6:**
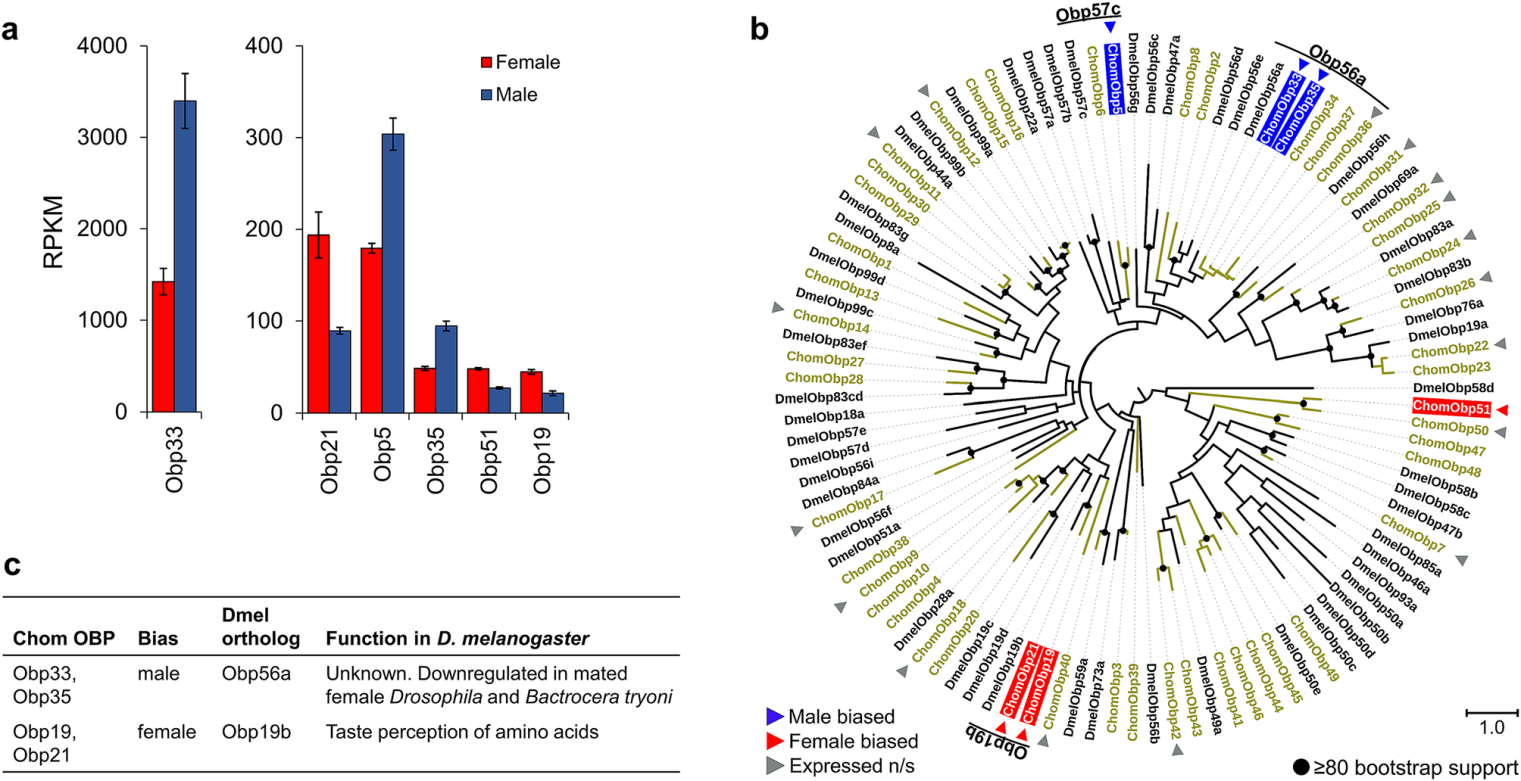
Sex-biased expression of OBPs in *C. hominivorax* antennae. (**a**) Twenty-three OBPs were expressed in the antennae, of which six were DE between sexes, three with male-biased expression and three with female-biased expression. Differential expression based on FDR adjusted p < 0.05. (**b**) Of the six DE OBPs, five are related to *D. melanogaster* proteins based on the estimated OBP phylogeny. Figures were generated using the interactive Tree of Life (iTOL) v4 software^36^ (https://itol.embl.de/). (**c**) In *D. melanogaster,* Obp19b is involved with taste perception of amino acids^44^. The chemosensory role of the remaining DE OBPs is largely unknown.

Three of the 21 IRs expressed in the antennae were DE between sexes, all being female-biased (Fig. 5a). The estimated IR phylogeny suggests that these are closely related to their namesake in *D. melanogaster* (Fig. 5b). In *D. melanogaster*, *Ir75a* detects several carboxylic acids (acetic, butyric and propionic)^40^, while *Ir76a* detects amines such as pyrrolidine and phenylethylamine^41^ (Fig. 5c). The types of IRs expressed in *C. hominivorax* were similar to those expressed in *D. melanogaster* antennae, with the following exceptions. *ChomIr31a*, *ChomIr60a* and *ChomIr68a* were not expressed in *C. hominivorax* but are commonly expressed in *D. melanogaster*, while *ChomIr10a* was expressed in *C. hominivorax* but is not typically expressed in *D. melanogaster* antennae^42^. Of the OBPs expressed in the antennae, six were DE between the sexes, with three being female-biased and three being male-biased (Fig. 6a). Five of these are closely related to *D. melanogaster* OBPs based on the estimated phylogeny (Fig. 6b). Thus far, a role in olfaction has been described only for *DmelObp49a*, where it was shown to be associated with bitter taste inhibition to sugar feeding^43^ (Fig. 6c).

None of the 15 GRs expressed in *C. hominivorax* antennae were DE between sexes. The antennal GRs included three putative CO_2_ receptors, of which two (*ChomGr2* and *ChomGr3*) were among the most highly expressed GRs, while the third (*ChomGr1*) was expressed at a much lower level (Fig. S1a,b). All six putative sugar receptors (*ChomGr4*-*Gr9*) were expressed in the antennae (Fig. S1a,b). The estimated GR phylogeny suggests *ChomGr25* is closely related to *DmelGr57a* (Fig. S1b), which is associated with bitter taste in *D. melanogaster*^45^. These results are similar to findings in *D. melanogaster* in which two CO_2_ receptors and five sugar receptors were expressed in the antennae^46^. Function of the remaining five GRs is difficult to predict due to their divergence from *D. melanogaster* GRs.

## Discussion

We utilized a multifaceted approach to address knowledge gaps in screwworm fly olfactory physiology, with the aim of developing more effective and specific attractants for screwworm fly control strategies. This was the first study of its kind in which olfactory responses of mated and unmated female *C. hominivorax* or *C. macellaria* were compared. Furthermore, we report for the first time quantitative analysis of the antennal transcriptome in male and female *C. hominivorax*.

Antennal responses of mated and unmated female *C. hominivorax* to *swormlure-4* constituents were similar, except for phenol, which elicited greater EAG signal in unmated females. None of the tested chemicals elicited a greater response in mated females. This is somewhat unexpected since the *swormlure* constituents were mostly derived from the analysis of bovine blood and odors associated with decomposition of meat protein and fatty acids representing an oviposition substrate for screwworm flies^15^. In *C. macellaria*, however, dimethyl disulfide elicited a larger physiological response in mated flies compared to unmated females. An additional use for a lure attracting primarily mated females, other than surveillance, is the potential for the development of a lethal oviposition trap for population control. The utility of such a trap has been demonstrated for mosquito species^47^, and remains to be for explored for control of screwworm.

There were no significant differences in the antennal responses to the tested constituents when males were compared with mated and unmated female *C. hominivorax* or *C. macellaria*. This is in contrast to a report where *p*-cresol (4-methylphenol) and dimethyl disulfide elicited a greater physiological response in males^48^. Field studies have differed in their proportion of males and females collected in traps baited with *swormlure*-*4*, with studies ranging from no sex bias^49^ to strong female-bias^8,29,50^. It was demonstrated during the development of the first synthetic lure (*swormlure*) that attraction was greater to the blend than to the individual constituents^15^, therefore, it is possible that the blend (*swormlure-4*) elicits a sex biased response while the individual constituents do not.

Multivariate analysis (PCA) suggests *C. hominivorax* and *C. macellaria* differed in their antennal responses to the *swormlure* constituents, even though sex or physiological state (virgin vs. gravid) differences were not that apparent within a species. However, this should be interpreted with caution: EAG studies for *C. macellaria* were performed in the laboratory at the University of Kentucky wherein optimum recording condition, such as stable temperature, humidity and noise-free electrical contacts are observed. Antennal recordings of *C. hominivorax* conducted at COPEG didn’t have all these optimal conditions. Consequently, we avoided direct comparison of EAG responses of *C. hominivorax* with *C. macellaria*. Due to their close evolutionary relationship yet different oviposition substrates preference (live animal wounds vs carrion or necrotic tissue), comparative chemical ecology and molecular olfaction studies of *C. hominivorax* and *C. macellaria* could improve the species-specificity of synthetic attractants for the screwworm fly and yield insights into the evolution of parasitism in Cochliomyia in the future.

In contrast to our EAG analysis of *C. hominivorax* in which there were no major sex-specific differences in antennal responses elicited by the tested chemicals, antennal transcriptome analysis revealed 29% of the expressed ORs as sex-biased. The simplest explanation for the disparity between EAG responses and gene expression analysis is that the EAGs are only proximate summation of the individual olfactory receptor neuron (ORN) responses^51^, and do not faithfully reflect the response of all chemosensory genes. Recent evidence also suggests variation in the transcriptional profiles of the chemosensory genes in natural mosquito populations^52^. Another likely explanation for absence of any sex-based correlation between the transcriptional profiles (Fig. 3) and EAG responses (Fig. 2) is that the ORs that are major contributors for the sexually dimorphic transcription profiles do not significantly contribute to the EAG amplitudes measured for the *swormlure-4* constituents tested here. Indeed conventional EAG measurements are largely the summed responses from basiconic sensilla, and underrepresent the contribution of trichoid sensilla^51,53^.

Our phylogenetic analysis of the olfactory genes groups nine of the 16 sex-biased *C. hominivorax* ORs with *D. melanogaster* receptors associated with pheromone sensing^35,38,39^. In *Drosophila*, trichoid sensilla respond to pheromone related odors, whereas most of the food/host odors are detected by basiconic sensilla^54,55^. *ChomOr63* (male-biased) is closely related to *DmelOr85f*, which was shown to be associated with innate avoidance of actinidine and nepetalactol, volatiles associated with the *Drosophila* parasitoids in the genus *Leptopilina*^37^ (Fig. 4b,c). *ChomOr19* and *ChomOr62* (female-biased) are closely related to *DmelOr7a* and *DmelOr88a*, respectively (Fig. 4b). *DmelOr7a* was shown to be necessary for aggregation and oviposition site selection via *9*-tricosine signaling^39^, while *DmelOr88a* was required for short-range attraction of both sexes to the fly-produced odors methyl laurate, methyl myristate and methyl palmitate^38^ (Fig. 4c). The remaining seven sex biased ORs had either no close phylogenetic relationship or their function has not yet been well characterized in *D. melanogaster*. Previous studies have established a role for chemical sexual communication in *C. hominivorax,* implicating male-produced^56^ and female-produced^57,58^ substrates such as cuticular hydrocarbons (CHCs). However, despite the identification of several CHCs^59–61^, their behavioral significance has yet to be realized. With the availability screwworm genome^30^, and specifically the development of genetic knockout techniques in these flies such as CRISPR/Cas9^62^, we aim to understand these behaviors driven by chemosensory genes. Identification of the ligands for the male- and female-biased receptors we describe here could identify chemicals for the development of sex-specific attractants.

The contribution of antennal IR expression on the EAG responses is unclear. These genes are primarily expressed in coeloconic sensilla^41,42^. Two of the three female-biased and highly-expressed IRs in the *C. hominivorax* antennal transcriptome (*ChomIr75a*-2 and *ChomIr75a*-3) are closely related to *DmelIr75a*, which responds to the short chain carboxylic acids^40^. Electroantennograms represent an approximate global olfactory responses from the antenna depicting the overall response dynamics and amplitude resulting from multiple ORNs expressing different families of chemosensory receptor genes, such as ORs, IRs or GRs^63,64^. As we noted earlier, acetic acid and butyric acid induced occasional hyperpolarizations. Short chain carboxylic acids, especially at high doses, often elicit upward deflection in EAG recordings across a range of insects^65^, and this has been ascribed to the acidity of these compounds and/or sometimes, as an artifact electrode potentials resulting from interaction of acids with the electrodes^66^. Our conclusion of the acid induced hyperpolarization, based on multiple measurements across both sexs and species, is that the measured signals are not artifacts.

Monitoring for the presence of *C. hominivorax* is an important component of screwworm control and eradication programs^67^. Though various measures are utilized for monitoring, synthetic attractants (e.g. *swormlure*) are particularly appealing due to the ease and expense at which they can be employed compared to alternative attractants, such as rotten beef liver. However, gaps in our knowledge have prevented advancement of synthetic attractant strategies for the screwworm fly. Improvement in species specificity and the development of a male-specific and gravid-female specific attractants is highly desirable. Here, we used a multifaceted approach in which we integrated neurophysiology, molecular and phylogenetic methods to gain a better understanding of *C. hominivorax* biology, toward the development of novel attractants for control of screwworm. We are in the process of functional characterization of the promising chemosensory genes we identified here based on the differential expression pattern and their phylogenetic relationship to the *D. melanogaster* orthologues that have been fully characterized. Prospecting the cognate ligands from the natural milieus such as open wounds and conspecifics will lead to the isolation and identification of chemostimuli that will be tested for the behavioral response, and eventually as odor baits in the surveillance traps.

## Methods

### Flies

The J06 strain of *C. hominivorax* was used for antennal transcriptome analysis and electroantennography (EAG). The J06 wild type strain of *C. hominivorax* was originally collected in Jamaica in 2006, and this strain was being used at the COPEG in Panama for SIT release at the time of the study.Flies were reared at the COPEG biosecurity plant in Panama using methods described previously^68^. *C. macellaria* used for EAG were originally collected in 2011 from a pig carcass located at the North Carolina State University (NCSU) Poultry field laboratory in Raleigh, North Carolina (35.724 N, − 76.687 W), and are maintained at North Carolina State University at 27 °C with 16:8 light dark cycle. Adult flies were provisioned with granulated sugar and water ad libitum. Weekly, adult fly cages were provided with 10 g of raw beef liver as a protein source to optimize ovary development. Two-week-old flies were provided a cup containing 10 g of raw liver loosely covered with a moist paper towel to encourage oviposition. Collected eggs were partitioned into pea sized (10 mm) allotments for transfer to the prepared larval medium consisting of 750 ml dry Purina Cat Chow Complete (Nestle Purina PetCare Company, St. Louis, MO) soaked in 400 ml of water. In a well-ventilated room, the larval medium bucket was placed on a rack at a 35° angle, the eggs added and the bucket covered with a screened lid. Pupae were harvested in 7 to 8 days.

### EAG recordings

Mounting of *C. hominivorax* for EAG was conducted by excising the head of the fly (3–6 days post-eclosion) with microscissors and placing it on an antenna holder (Syntech, Germany) with the base of the head on one side and the tip of both antennae contacting the other. To improve electrical conduction and reduce desiccation, Spectra 360 Electrode Gel (Parker Laboratories) was applied at the contacts between the holder and the fly (base of head and tip of antennae). We note that due to biosecurity measures, these recordings could only be conducted inside the COPEG production facility, which is not optimal for fine electrophysiological measurements. Mounting of *C. macellaria* for EAG was similar to fruit flies recordings we routinely employ^69^ with a few modifications. A fly (3–8 days post-eclosion) was inserted in a 1000 μl pipette tip (USA Scientific Inc.) with approximately one-third of the head protruding from the tip. The tips of both antennae were placed in a 1 mm borosilicate capillary (World Precision Instruments, USA) containing sensillum lymph ringer^65^, while a ground was placed into the eye. Signals were amplified and recorded using an IDAC2-USB box (Syntech) with a 0.05 Hz low cutoff. Each recording was a total of 10 s in length with a set 1 s pre-trigger. A CS-55 Stimulus Controller (Syntech) was used to supply a charcoal filtered and humidified continuous air stream (0.8 l/min) delivered via a Teflon tube. A stimulus pulse (0.8 l/min) was added to the air stream for 0.5 s. Recordings were analyzed with EAG Pro version 1.1 software (Syntech).

Chemicals used for stimulus and solvent were of high purity: 1-octen-3-ol (98% purity) 2-butanol (analytical standard), 2-methyl-1-propanol (analytical standard), acetic acid (99.5%), benzoic acid (analytical standard), butyric acid (analytical standard), dimethyl disulfide (analytical standard), dimethyl trisulfide (98%), indole (98%), p-cresol (≥ 99%), phenol (≥ 99%) and valeric acid (analytical standard). Chemicals were dissolved in dichloromethane (DCM ≥ 98%) to make a stock solution of 100 mg/ml (− 1). A 1:10 dilution (− 2) of the stock solution in DCM was used as stimulus. A 20 µl of stimulus solution was loaded onto a filter paper strip. After allowing the solvent to evaporate for 10–15 s, the strip was placed in a 5 ml polypropylene syringe for delivery onto the EAG preparation. Each recording was first baseline normalized, then smoothed with a 200 ms running average, amplitude determined and the value of the appropriate blank (solvent) control subtracted.

The antennal response for each compound is represented as the proportion of the summed responses of each individual to all compounds. Statistical tests for difference in antennal response between sexes and/or physiological state (virgin and gravid), and principal component analysis (PCA) were conducted using SigmaPlot v14.0. Data were initially assessed and passed tests for normality (Shapiro–Wilk) and equal variance (Brown-Forsythe) prior to implementing a one-way ANOVA and Tukey post-hoc test (when ANOVA p-value < 0.05). Principal component analysis was conducted with the covariance method using the average eigenvalue for component selection. The Mann–Whitney rank sum test was used to test for difference between principal component scores for PC1 in Fig. 2d.

### Antennal transcriptome analysis

The methods used for extraction and quantification of total RNA, and preparation of cDNA libraries are described in Scott et al.^30^. In brief, antennal RNA was extracted from frozen samples, and RNA integrity, purity, and concentration were assessed using an Agilent 2100 Bioanalyzer. Purification of mRNA was performed using the oligo-dT beads provided in the NEBNExt Poly (A) mRNA Magnetic Isolation Module. Complementary DNA libraries for Illumina sequencing were constructed using the NEBNext Ultra Directional RNA Library Prep Kit (NEB) and NEBNext Mulitplex Oligos for Illumina (NEB) using the manufacturer-specified protocol. The amplified library fragments were purified and checked for quality and final concentration using an Agilent 2200 Tapestation with a High Sensitivity DNA chip (Agilent Technologies, USA) and a Qubit fluorometer (ThermoFisher, USA). The quantified cDNA libraries were pooled in equimolar amounts for clustering and sequencing on an Illumina HiSeq 2500 DNA sequencer, utilizing a 125 bp single-end sequencing reagent kit (Illumina, USA).

Sequencing depth ranged from 8.74 to 13.06 million mapped reads per library (mean = 10.45). Sequence reads were mapped to the *C. hominivorax* genome assembly using Hisat2 v2.1.0^70^. Read counts were obtained using HTSeq-count (*union* mode) in HTSeq v0.11.2^71^. The quasi-likelihood F-test and false discovery rate (fdr) correction for multiple tests in EdgeR was used for differential expression^72,73^. Data were converted from CPM (counts per million reads) to RPKM (reads per kilo base per million mapped reads) following statistical analysis. SigmaPlot v14.0 was used for PCA of chemosensory gene expression (RPKM) using the correlation method and the average eigenvalue for component selection.

### RT-qPCR validation of candidate *C. hominivorax* genes

RNA was isolated following the Direct-zol protocol (Zymo Research, Irvine, CA) from five pairs of male and female adult fly antenna that were shipped from COPEG, Panama in RNA-shield (Zymo Research, Irvine, CA). The quantity and quality of the isolated RNA was assessed using a NanoDrop. First-strand cDNA was synthesized from 300 ng of total RNA per sample using the Superscript II protocol (Thermo Fisher, Carlsbad, CA). cDNA for each sample was diluted to 20 ng/µl for subsequent qPCR reactions. Actin and *RPS17* served as endogenous control genes for qPCR since these were shown to be the top and most stably expressed genes within the members of Calliphoridae^74^. Each 10 µl PCR reaction included 2 µl of cDNA template (20 ng/µl), 5 µl of SYBR GRN master mix, 1 µl of primer (0.5 µl F and 0.5 µl R) and 2 µl of sterile water. PCR cycling conditions were 95 °C (3 min) and 40 cycles of 95 °C (10 s) and 60 °C for 30 s. For each gene, a standard curve was derived to assess the amplification efficiency of the primers, melting curve to assess the specificity of the primers and included three biological replicates. Differential expression analysis was based on the well establish method^75^.

### Phylogenetic analysis of chemosensory genes

Phylogenetic relationships among *C. hominivorax*^30^ and *D. melanogaster*^40,76,77^ chemosensory genes were estimated by first conducting a multiple protein alignment for each gene family using default parameters in ClustalX v2.1^78^. Alignments were visually inspected and then used for maximum likelihood estimation in RAxML v8.2.11 with the PROTGAMMAAUTO model option and 500 bootstrap replications^79^. Figures were generated using the interactive Tree of Life (iTOL) v4 software^36^ (https://itol.embl.de/).

## Supplementary information

Supplementary Figure S1.

Supplementary Table S1.
