# Supplementary material for: Physiological and molecular correlates of the screwworm fly attraction to wound and animal odors
Source: Sci Rep. 2020 Nov 27;10:20771. doi: 10.1038/s41598-020-77541-w (PMC7695851; doi:10.1038/s41598-020-77541-w)
Supplement: Supplementary file 2 — Supplementary Table S1. [file 41598_2020_77541_MOESM2_ESM.docx]

**Supplemental material for**

**“Physiological and molecular correlates of the screwworm fly attraction to wound and animal odors”**

Paul V. Hickner^1^, Omprakash Mittapalli^1^, Anjana Subramoniam^1^, Agustin Sagel^2^, Wes Watson^3^, Maxwell J. Scott^3^, Alex P. Arp^4^, Adalberto A Pérez de León^4^, and Zainulabeuddin Syed^1*^

**Figure S1**


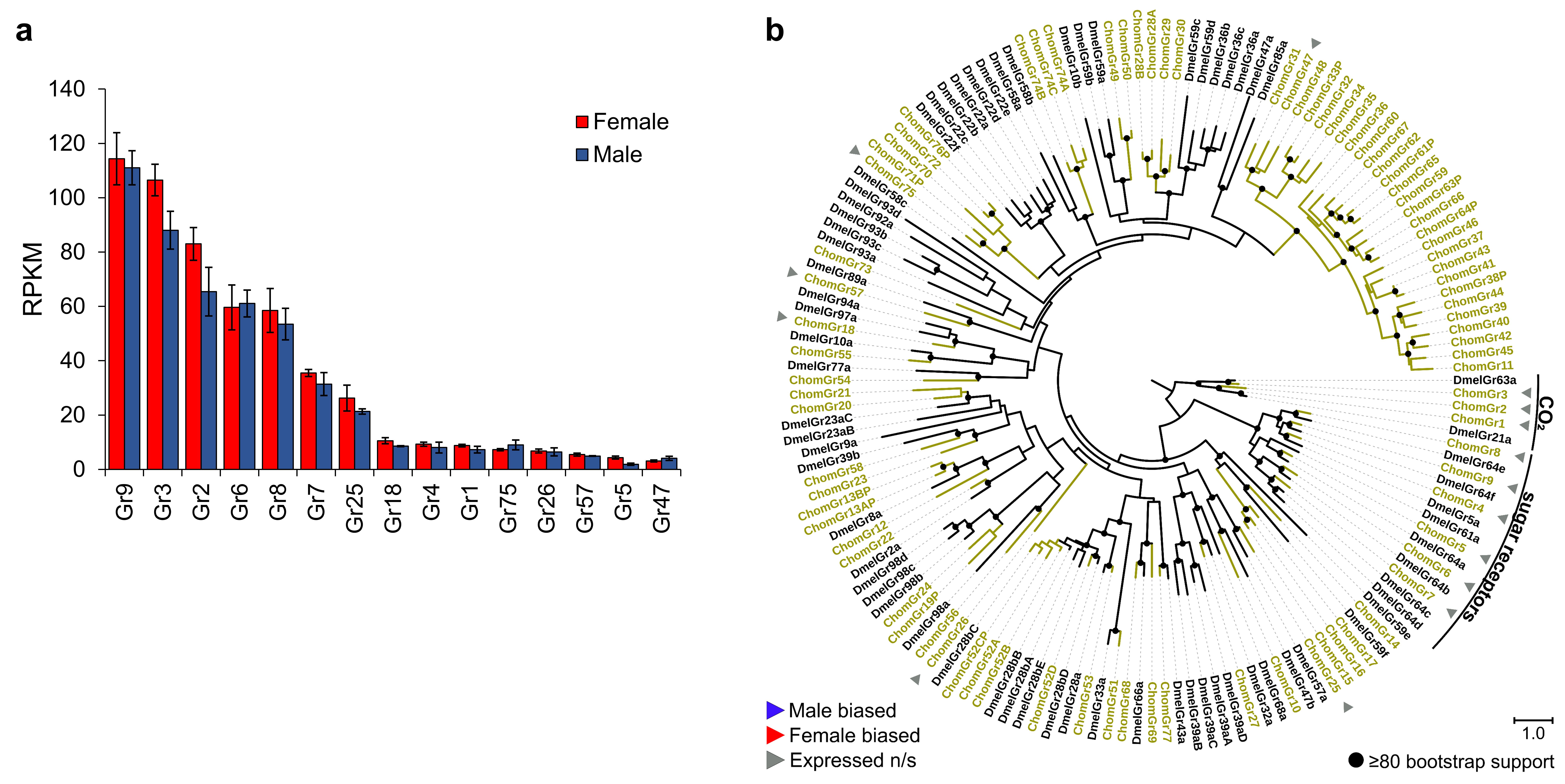


**Figure S1.** Expression of GRs in *C. hominivorax* antennae. **a)** Fifteen GRs were expressed in the antennae, of which none were DE between sexes. **b)** Of the expressed GRs, three are related to CO_2_ receptors and six are related to sugar receptors in D. melanogaster. Figures were generated using the interactive Tree of Life (iTOL) v4 software (https://itol.embl.de/).
